# Life cycle stage practices and strategies for circular economy: assessment in construction and demolition industry of an emerging economy

**DOI:** 10.1007/s11356-022-21470-w

**Published:** 2022-06-24

**Authors:** Richard Asante, Daniel Faibil, Martin Agyemang, Sharffudin Ahmed Khan

**Affiliations:** 1 Department of Civil Engineering, Fujian University of Technology, Fuzhou, Fujian Province People’s Republic of China; 2 School of Economics and Management, Beijing Institute of Technology, Beijing, People’s Republic of China; 3 Cardiff Business School, Cardiff University, Cardiff, UK; 4 Industrial Systems Engineering, Faculty of Engineering and Applied Sciences, Regina, SK Canada

**Keywords:** Circular economy, Life cycle, Building industry, Strategy, Implementation, Emerging economy, 6R framework

## Abstract

The strategic implementation of circular economy (CE) practices in the construction and demolition (C&D) industry is critical for achieving environmental sustainability goals. Understanding CE practices based on reduce, reuse, recycle, recover, remanufacture, and redesign (6R) principles from the perspective of the whole life cycle can promote the implementation of CE practices in the C&D industry. However, studies that shed light on this subject especially in emerging economies are generally lacking. This study contributes to filling this gap by using a three-phase methodology consisting of a literature review and a hybrid best–worst method and grey relational analysis to give insights into practices and strategies to prioritize CE practice implementation. Specifically, the paper focuses on identifying CE practices based on 6R principles, the significance of the identified CE practices, and understanding how to prioritize the implementation of the significant CE practices. The study’s findings established that implementing CE practices based on reduce and recover principles at the design stage contributes significantly to environmental sustainability. Additionally, the study highlights the relevance of both bottom-up and top-down approaches in the implementation of CE practices.

**Supplementary Information:**

The online version contains supplementary material available at 10.1007/s11356-022-21470-w.

## Introduction

The construction and demolition (C&D) industry is responsible for significant environmental effects throughout the whole building life cycle (López Ruiz et al. 2020). The industry is under significant pressure to implement sustainable practices (Yu et al. 2013; Esa et al. 2017). Circular economy (CE) which has received attention from many stakeholders and researchers worldwide (Merli et al. 2018) is regarded as the potential solution to the industry’s sustainability issues (Lei et al. 2021). However, many stakeholders in the C&D industry still lack knowledge on how to implement CE practices (Antwi-Afari et al. 2021).

Transition to environmental sustainability requires CE practice implementation across the whole life cycle (Mura et al. 2020). Despite CE practice implementation across the whole life cycle of buildings drawing much interest in recent times (Lei et al. 2021), studies that propose a unified approach in the C&D industry of emerging economies are rare (López Ruiz et al. 2020; Benachio et al. 2020). Only a few studies such as Benachio et al. (2020) and Guerra et al. (2021) proposed a unified approach to guide CE practice implementation across the whole life cycle of buildings. However, the authors did not provide a hierarchical analysis of the identified CE practices to facilitate the strategic implementation in the C&D industry especially for emerging economies where firms lack sufficient resources (Agyemang et al. 2020; Faibil et al. 2021; Asante et al. 2022).

Implementation of CE practices based on reduce, reuse, recycle (Huang et al. 2018), recover (Yang et al. 2017), remanufacture, and redesign (6R) principles (Jawahir and Bradley 2016) has shown better results in terms of general performance all over the world (Govindan and Hasanagic 2018). Strategic implementation of CE practices in emerging economies that draws insight from 6R principles and the perspective of the whole life cycle is generally lacking in many sustainability studies on the C&D industry (López Ruiz et al. 2020). The study contributes to this research gap associated with CE implementation in emerging economies by focusing on the Ghanaian C&D industry. The Ghanaian C&D industry has given less attention to the environmental sustainability of its activities (Agyekum et al. 2020). Government and stakeholders’ environmental sustainability strategies have often been described as non-cohesive (Ahmed et al. 2014) and characterized as ineffective (Ofori et al. 2015).

Therefore, drawing insight from 6R principles, the study aims to develop strategies to inform and guide stakeholders in emerging economies particularly top management leadership to facilitate CE practice implementation over the whole life cycle of buildings. The research aim is guided by the following research questions (RQ):

- RQ1: what is the significance of identified CE practices for implementation?
- RQ2: how can CE practices be strategically implemented in the Ghanaian C&D industry?

## Research background

### Circular economy (CE) in the Ghanaian construction and demolition (C&D) industry

Insufficient infrastructure remains a major issue in Ghana, and many emerging economies as population growth and urbanization increase continue to outstrip infrastructural development (Ansah et al. 2020). The infrastructural deficit has caused an increase in construction activities (Zhang et al. 2015). The activities of the Ghanaian C&D industry are vital in achieving national socio-economic development goals (Anaman and Osei-Amponsah 2007). The industry contributed 13.7% to the GDP in 2017 (Ghana Statistical Service 2018). The four main stakeholders in the industry are government, clients, contractors, and consultants (Dadzie et al. 2012; Donkoh and Aboagye-Nimo 2017). Generally, the industry is characterized by an insufficient skilled workforce, heavy reliance on labor-intensive methods, and huge informal sector participation (Boadu et al. 2020).

The concept of CE is new in the Ghanaian C&D industry (Keesman 2019) and other emerging economies in Africa (Rademaekers et al. 2020). Many firms in the Ghanaian C&D industry and other emerging economies in Africa still adopt a linear model of practices (Djokoto et al. 2014; Rademaekers et al. 2020). Only a few emerging economies in Africa such as Ethiopia, Kenya, and Rwanda have developed strategies to facilitate the implementation of CE practices (Desmond and Asamba 2019). The C&D industry in Ghana and other emerging economies in Africa activities has increased resource consumption and environmental impact (Ametepey and Ansah 2014; Ragossnig 2020; Mhlanga et al. 2021).

Existing indigenous building practices (Kpamma et al. 2017) such as Zabur adobe building technique (Gruber and Datta 2021), burnt clay brick construction (Baiden et al. 2014), and others have been identified to support the transition to CE. However, the non-availability of standards, inability to satisfy modem design forms, and psychological resentment have affected the adoption of indigenous building practices in Africa (Acheampong et al. 2014).

Additionally, most industries in emerging economies adopt a bottom-up approach in the transition toward CE (Agyemang et al. 2019; Moktadir et al. 2018). The bottom-up approach involves a collaborative effort from individual firms, environmental organizations, and civil society toward the transition to CE (Brown and Stone 2007; Naustdalslid 2014). On the contrary, CE practice implementation in the Ghanaian C&D industry is based on the top-down approach. In the top-down approach, governments (central, regional, and municipal) formulate strategies and policies (Prendeville et al. 2018; Zhao 2020) which serve as a legal framework for CE transition. For example, the Government of Ghana has also been playing a leading role in the development of policy frameworks, including the Environmental Fiscal Reform Policy, National Climate Change Adaptation Strategy, and many others to address sustainability concerns (Hemkhaus et al. 2020).

### The “6R” principles

Table 1 shows the definition of the 6R principles used in this study.

**Table 1:** 6R principle definition

6R principles	Definition	Source
Reduce	Focuses on sustainable consumption of resources and energy at the design, construction, and operation stage	US Environmental Protection Agency (2008); Zhang et al. (2013a)
Reuse	Involves the reuse of components after their first life cycle or other subsequent life cycles to reduce the use of virgin materials in the production of a new component	Jawahir and Bradley (2016)
Recycle	Process of converting components that cannot be restored into their original state into new materials or components	Zhang et al. (2013a); Jawahir and Bradley (2016)
Recover	Involves the process of collecting materials at the end-of-life stage and then disassembling, sorting, and cleaning for use	Joshi et al. (2006)
Remanufacture	Involves the re-processing of already used components for restoration to their original state or a like-new form through the reuse of as many parts as possible without loss of functionality	Jawahir and Bradley (2016)
Redesign	Involves manufacturing or remanufacturing next-generation components using materials recovered from the previous life cycle or previous generation of components	Zhang et al. (2013b); Jawahir and Bradley (2016)

### Life cycle stages in the construction and demolition (C&D) industry

There is no consensus on life cycle categorizations in the C&D industry from CE perspective. Different authors such as Lu et al. (2021), Esa et al. (2017), Shen et al. (2007), Benachio et al. (2020), López Ruiz et al. (2020), Guerra et al. (2021), and others have categorized life cycle differently. Therefore, to determine the appropriate life cycle stage applicable to this study, several discussions among ten purposively sampled experts were conducted to ascertain the expert’s view on the identified life cycle categorizations in Table 2. The experts also characterize the life cycle stage-related CE practices identified from studies such as Adams et al. (2017), López Ruiz et al. (2020), Benachio et al. (2020), and others based on the 6R principles. A summary of the characterization of life cycle stage-related CE practices identified by the ten experts is presented in Table 3.

**Table 2:** Life cycle categorization summary

Source	Categorization
Lu et al. (2021)	Design, construction, operation, maintenance, and demolition
Esa et al. (2017)	Planning, designing, procurement, construction, and demolition
Shen et al. (2007)	Inception, design, construction, operation, and demolition
Benachio et al. (2020)	Project design, material manufacture, construction, operation, and end of life
López Ruiz et al. (2020)	Pre-construction, construction, and building renovation, collection and distribution, end of life, and material recovery and production
Guerra et al. (2021)	Design, construction, and end of life

**Table 3:** Life cycle stage-related CE practices characterize based on 6R principles

	Life cycle stage			
6R principles	Design stage (includes LCS1)	Construction stage (LCS2)	Operation stage (LCS3)	End-of-life stage (LCS4)
Reduce (R1)	a. Design to improve the energy efficiency of buildings	a. Proper material storage location	a. Adoption of sustainable repair and maintenance practices	-
	b. Design to prevent the generation of waste	b. Adoption of sustainable construction method	b. Refurbish/renovate with reused/recycle material	-
	c. Make allowance for incentives for CE practices in the contract document	c. Resource optimization to minimize excessive use of resources on site	c. Efficient practices by end-user to reduce CO_2_ emissions and environmental footprint	-
	d. Design with sustainable local materials such as rammed earth and clay plaster	d. Minimize material stockholding	d. Efficient use of building to prevent rapid degradation of materials and elements	-
	e. Design to allow for the use of recycled materials	e. Integration of recycled materials	-	-
	f. Engagement of stakeholders to ensure the design conforms with CE principles	f. Proper management of the construction process	-	-
	g. Standardization of designs	g. Prevent double handling of materials	-	-
	h. Design to increase the lifespan	h. Peer review of designs by stakeholders on site	-	-
Reuse (R2)	a. Design for reuse of building elements, e.g., columns and doors	-	-	-
Recycle (R3)	-	a. Provide incentives for recycling of waste materials on site	-	a. Recycling of waste materials
	-	b. Encourage reuse of materials	-	-
Recover (R4)	a. Design for deconstruction to ensure efficient recovery of materials	a. Encourage prefabrication construction	-	a. Deconstruction to obtain maximum recovery of the components to be used
	b. Design for the adoption of prefabrication	-	-	b. Selective demolition
	c. Design for easy disassembly	-	-	c. Record of the life cycle performance of materials
	-	-	-	d. End-of-life audits of recovered materials
Remanufacture (R5)	-	-	-	a. Adaptive reuse of the whole or part of the redundant structure or material
Redesign (R6)	-	-	a. Evaluate the life cycle performance of redesigned components	-

Based on the outcome of the discussion among the ten experts, the life cycle categorization for this study in the context of the Ghanaian C&D industry are design stage (includes preliminary studies, project design, and procurement process), construction stage (includes all process from possession of site by a contractor to handing over the project to the client), operation stage (includes the operation of building, refurbishment/renovation, repair, and maintenance), and end-of-life stage (includes demolition, material recovery, and disposal). The life cycle categorization proposed in this study closely aligns with life cycle categorization in previous studies such as Lu et al. (2021), Shen et al. (2007), and Guerra et al. (2021).

## Methodology

A three-phase methodology approach which includes a literature review and a hybrid multi-criteria decision-making (MCDM) tool was employed to address the study’s research questions. MCDM represents a novel tool to measure CE practices and strategies to facilitate its implementation (dos Santos Gonçalves and Campos 2022). The hybrid MCDM approach comprises best–worst method (BWM) and grey relational analysis (GRA) methods. The BWM technique is selected to analyze and prioritize the relative weights of each life cycle stages. The BWM techniques require less number of pairwise comparisons. It provides realistic and reliable results, and it is straightforward and easy to compute (Chen et al. 2020; Kumar et al. 2020). The GRA method is integrated with the BWM technique to determine the intensity and rankings of all practices identified for CE implementation in the C&D industry. GRA technique is unlimited to the number of criteria for a study, and it’s easy to understand by decision-makers which expedites the decision-making process (Kuo et al. 2008; Song et al. 2017). The step for BWM and GRA is presented in Appendix B and C, respectively.

The experts identified the life cycle categorization applicable to this study. Subsequently, they characterized CE practices in Table 6 in Appendix A identified from extant literature and applicable to the Ghanaian C&D industry based on the 6R principles. The results from the survey are presented in Table 3.

### The hybrid best–worst method and grey rational analysis approach for this study

In the present study, the hybrid BWM-GRA technique is formulated to determine the weights and the ranking of the life cycle stages and related CE practices in the C&D industry. BWM was preferred among other forms of MCDM because it requires less pairwise comparison data and processes more consistent results (Rezaei 2015a, b). GRA was chosen because it neutralizes decision-making challenges such as uncertainties, fuzziness, and subjectivity (Deepanraj et al. 2017; Liao et al. 2017). The first of the two-stage approach involves enlisting all the life cycle stages and CE practices in the C&D industry based on the 6R principles. Four life cycle stages and thirty-four CE practices were identified and approved for the study through extensive literature review and experts’ views. Then, BWM questionnaires were structured and sent to experts for a pairwise comparison of the life cycle stages using a score scale between 1 and 9, as indicated in Tables 9 and 10 in Appendix F. The experts were asked to select the most important (*B*) and the least important (*W*) life cycle stages criteria over the other based on the rating scale given. The data obtained were analyzed using the BWM technique to calculate the criteria weight of all the life cycle stages for further analysis as shown in Table 4. The criteria weights obtained from the life cycle stages were integrated with the GRA ratings of the CE practices to determine the grey relation grade of the CE practices for subsequent rankings.

**Table 4:** Ranking of life cycle stages

Life cycle stages	Consistency ratio	Main weights	Ranking
LCS1	0.137	0.576	1
LCS2	0.137	0.152	2
LCS3	0.137	0.134	4
LCS4	0.137	0.137	3

**Fig. 1:**
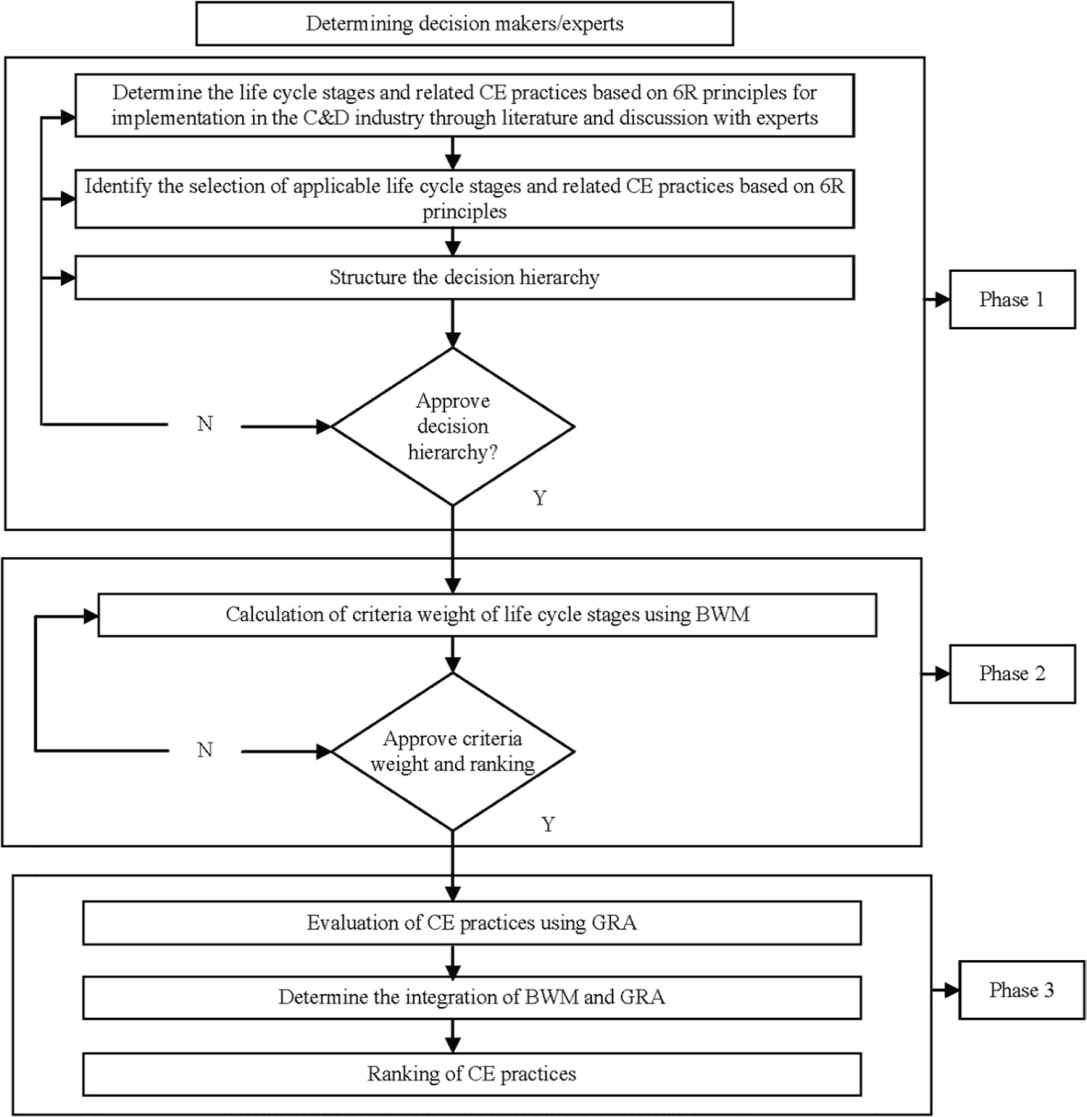
Three-phase methodology applied in the study

In the second stage, the experts rated all the thirty-four CE practices under each life cycle stage employing a score scale between 1 and 9, with 1 being least and 9 being the highest value. Then, the data generated were computed and normalized using the GRA technique as indicated in Table 22 in Appendix H. The normalized figures were then analyzed to determine the grey relation coefficient value shown in Table 23 in Appendix H. The grey relation coefficient values of the CE practices were integrated with the criteria weight of all the life cycle stages using Eq. (17) in Appendix D. Then, we calculated the grey relation grade for all the CE practices using Eq. (18) in Appendix D for the ranking of the CE practices as presented in Table 24 in Appendix H. The hybrid BWM-GRA approach applied in this study is summarized and presented in Appendix D. The methodology for this study is also summarized in Fig. 1.

## Results

### Background of the experts

The experts for the study were chosen primarily based on their knowledge about the objective of the study and their experience in the industry. The background for the experts is presented in Table 8 in Appendix E. Each expert had over 10 years of experience in the C&D industry.

### Criteria weight calculation using best–worst method (BMW)

The experts were first asked to determine the most and least important life cycle stage. They were subsequently asked to determine the preference of the most important life cycle stage over the others and preference of the others over the least important life cycle stage using a scale of 1–9. A summary of their preference is presented in Appendix F. A set of attributes comprising an initial pairwise comparison of CE practices by various experts is presented in Appendix G.

The results of the life cycle stages ranking indicate design stage (0.576) is the most significant. The ranking of other life cycle stages in order of importance includes construction stage (0.152), end-of-life stage (0.137), and operation stage (0.134), respectively.

### Ranking of circular economy (CE) practices using grey rational analysis (GRA)

The GRA method was used in the calculation of the ranking of the CE practices identified in the study. The experts were also asked to rate all the CE practices identified under the four main life cycle stages using a scale of 1–9, as shown in Tables 12, 13, 14, 15, 16, 17, 18, 19, 20, and 21 in Appendix G. Grey relational grade was calculated based on the steps outlined in Appendix D of the study. The local and global ranking of the CE practices identified under each life cycle stage was ranked according to their weights. The outcome of the global ranking was used to determine the significance of each 6R principle. The results of the rankings are presented in Table 5. The findings of the study were discussed with the ten experts. The experts suggested how stakeholders such as government, firms, NGOs, and professional bodies, among others, in the industry, contribute to the implementation of the four most significant CE practices to support the adoption of the relevant 6R principles. The experts also suggested what stakeholders can or need to do to support the implementation of the most significant CE practices.

**Table 5:** Local and global ranking of CE practices

	Life cycle stages															
6R	LCS1				LCS2				LCS1				LCS1			
	CE practices	Grey grades	Local rank	Global rank	CE practices	Grey grades	Local rank	Global rank	CE practices	Grey grades	Local rank	Global rank	CE practices	Grey grades	Local rank	Global rank
R1	LCS1R1a	0.576	1	1	LCS2R1a	0.059	7	19	LCS3R1a	0.045	4	23	-			
	LCS1R1b	0.254	7	7	LCS2R1b	0.152	1	9	LCS3R1b	0.045	4	23	-			
	LCS1R1c	0.192	8	8	LCS2R1c	0.082	4	14	LCS3R1c	0.134	1	11	-			
	LCS1R1d	0.334	5	5	LCS2R1d	0.051	9	21	LCS3R1d	0.080	2	15	-			
	LCS1R1e	0.302	6	6	LCS2R1e	0.071	6	18	-				-			
	LCS1R1f	0.302	6	6	LCS2R1f	0.089	3	13	-				-			
	LCS1R1g	0.453	3	3	LCS2R1g	0.051	9	21	-				-			
	LCS1R1h	0.576	1	1	LCS2R1h	0.076	5	16	-				-			
R2	LCS1R2a	0.373	4	4	-				-				-			
R3	-				LCS2R3a	0.059	7	19	-				LCS4R3a	0.137	4	10
	-				LCS2R3b	0.133	2	12	-				-			
R4	LCS1R4a	0.373	4	4	LCS2R4a	0.053	8	20	-				LCS4R4a	0.059	3	19
	LCS1R4b	0.334	5	5	-				-				LCS4R4b	0.073	2	17
	LCS1R4c	0.488	2	2	-				-				LCS4R4c	0.137	1	10
	-				-				-				LCS4R4d	0.073	2	17
R5	-				-				-				LCS4R5a	0.046	4	22
R6	-				-				LCS3R6a	0.071	3	18	-			

**Fig. 2:**
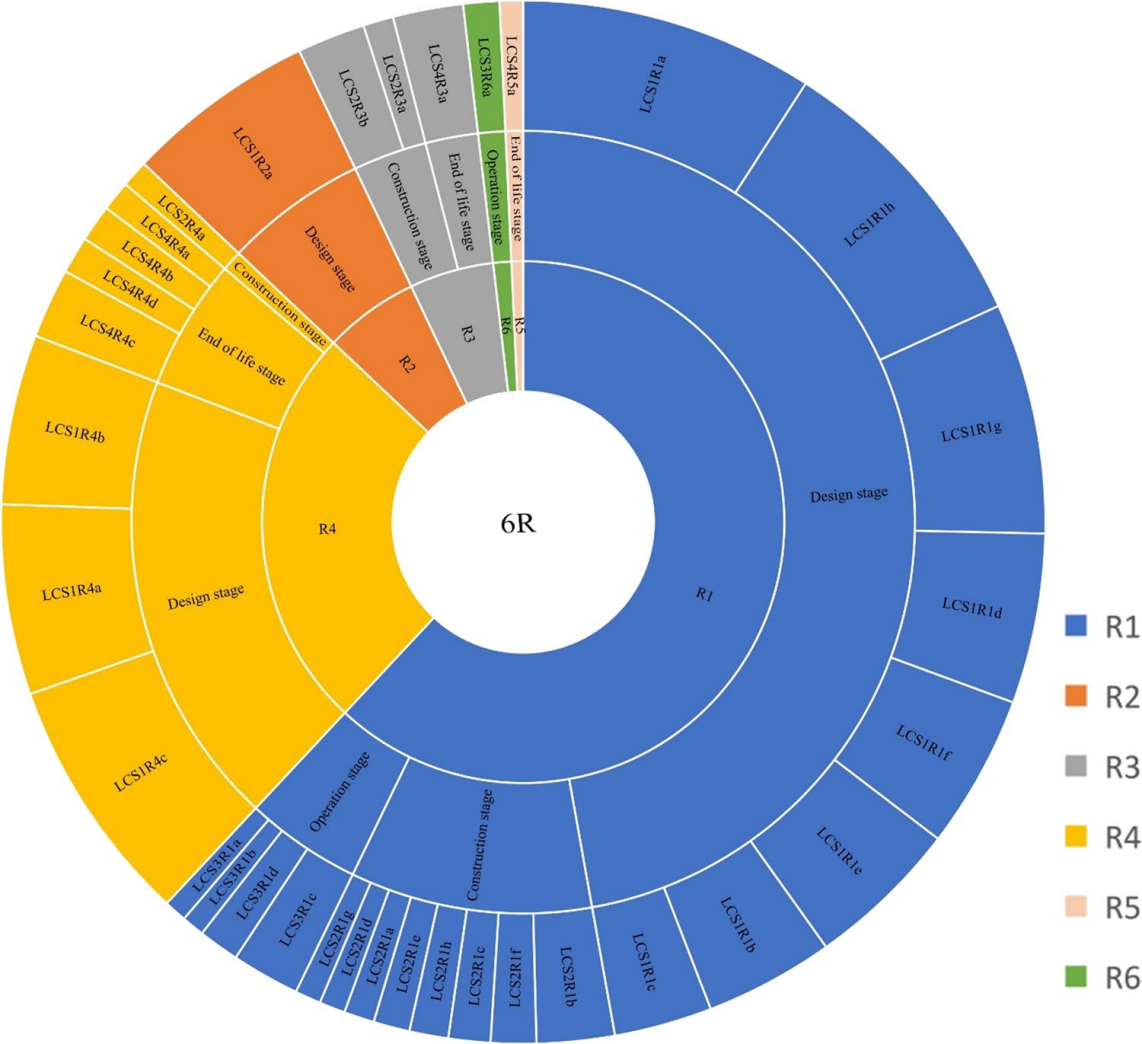
Global ranking of CE practices (data source, Table 5)

Based on the global ranking of CE practices presented in Table 5 and Fig. 2, all the CE practices under design stage were ranked as the most significant CE practices among the thirty-four CE practices identified in the study. The most prominent among them includes design to improve the energy efficiency of buildings (0.576), design to increase the lifespan (0.576), design for disassembly (0.488), and the standardization of designs (0.453). According to the most prominent CE practices, reduce and recover principles are the most significant among the 6R principles.

Many firms in the Ghanaian C&D industry adopt energy-efficient design techniques to improve the efficient use of natural ventilation and daylighting in buildings. Some leading firms adopt energy-efficient techniques such as passive cooling, solar thermal water system (The Architect’s Newsletter 2020), green roof system, and passive solar technique (Mensah et al. 2017; Asman et al. 2019) as strategies to support the adoption of reduce principle. NGOs, professional bodies, and individual firms such as the International Finance Corporation (IFC), Ghana Green Building Council, Ghana Institute of Architects (GIA), and Yecham Property Consult have also made significant contributions in terms of providing training and technical support to stakeholders in the Ghanaian C&D industry to improve the energy efficiency of buildings (Ankiilu, 2019; Ghana Green Building Summit 2020; International Finance Corporation 2017). Ghana Standard Authority (government state institution), together with professional bodies in the industry, has developed Ghana Building Code (GS1207) to support and enforce the adoption of energy-efficient design techniques in the C&D industry.

Among the four significant CE practices, design to increase the life span is the most common practice in the Ghanaian C&D industry. However, the common implementation may be primarily for economic incentives rather than environmental sustainability concerns (Masi et al. 2018). Firms in the Ghanaian C&D industry use materials and structural parts that comply with all relevant standards as techniques to increase the life span of buildings. The recent development of the Ghana Building Code (GS1207) to replace National Building Regulations, 1996 (LI1630), is one of the significant commitments from the government to ensure buildings are designed to increase their life span. Design for disassembly has the potential to support the recovery of materials at the end of life. However, insufficient professional knowledge and techniques for implementation of this practice (Djokoto et al. 2014; Ametepey et al. 2015) have hindered its integration into mainstream practices in the industry. Currently, there is no initiative by the government in promoting designs for disassembly. Thus, the government needs to develop policies or regulations to oblige firms to incorporate design for disassembly into their mainstream practices.

A standardized design reflects a firm’s environmental sustainability goals. Many real estate development firms such as Devtraco Plus Ltd., Laurus Development Partners, and others in the Ghanaian C&D industry have standardized design practices (Laurus Development Partners 2015; Devtraco Plus Ghana Ltd 2016) to facilitate sustainable energy and material consumption at the various life cycle stages. Currently, only the demand for environmentally friendly buildings by end-users has placed significant pressure on firms to develop a standardized design that reflects their environmental sustainability goals (Adjarko et al. 2016; Doku and Agarwal 2016). Therefore government, NGOs, and professional bodies in the Ghanaian C&D industry can also collaborate to pressurize firms to develop standardized design practices.

## Discussion

The finding of the study indicates stakeholders can facilitate the transition to CE by prioritizing CE practices at the design stage. Previous studies such as Akanbi et al. (2018) and De Magalhães et al. (2017) also acknowledge the crucial role of design stage in CE practice implementation. Prioritizing CE practices at the design stage that improves energy efficiency, increases life span, encourages disassembly, and standardizes design have the potential to improve the environmental sustainability of the C&D industry of emerging economies from the perspective of the whole life cycle.

The significance of the identified CE practices supports findings from previous studies such as Asman et al. (2019), Minunno et al. (2020), and Akanbi et al. (2018). Asman et al. (2019) emphasized the importance of design to improve the energy efficiency of buildings in the C&D industry. Minunno et al. (2020) and Akanbi et al. (2018) also acknowledge the significance of design for disassembly in CE implementation. Extant literature on CE implementation strategies focuses on reduce and reuse principle (Munaro et al. 2020). Among the 6R principles, many firms interested in CE practices implementation adopt recycle principle (Ghisellini and Ulgiati 2020). Nevertheless, the outcome of the study suggests prioritizing reduce and recover principles has the potential to facilitate the strategic implementation of CE practices.

### Theoretical implication

The study contributes to the strategic implementation of CE practices. First, in response to the research gap on the general lack of studies on a unified approach to guide the implementation of CE practices based on 6R principles in the C&D industry over the whole life cycle of buildings in emerging economies, we identified and characterize thirty-four CE practices into four life cycle stages and 6R principles. The study also makes theoretical contribution to CE practice implementation strategies from the perspective of the whole life cycle by emphasizing the important role of reduce and recover principles at the design stage. The study extends extant literature on the hierarchical analysis of life cycle stage-related CE practices by suggesting design to increase energy efficiency, design to increase the life span, design for disassembly, and standardization of design which should be prioritized to facilitate the transition toward environmental sustainability in the C&D industry of emerging economies.

Previous research such as Djokoto et al. (2014), Ofori et al. (2015), Ayarkwa et al. (2017), and Mensah et al. (2017) suggested top-down approaches which require government to provide financial support and develop policies or regulations for the implementation of CE practices. However, the findings of this study indicate many firms in the Ghanaian C&D industry that adopt the identified most significant CE practices, specifically at the design stage, do so without much government or public institutional support. Additionally, professional bodies, NGOs, and individual firms such as GIA, IFC, Ghana Green Building Council, and Yecham Property Consult, among others, have also made an important contribution to the implementation of the identified most significant CE practices in the Ghanaian C&D industry (Ankiilu, 2019; Ghana Green Building Summit 2020; International Finance Corporation 2017). Therefore, the study highlights the importance of both bottom-up and top-down approaches to prioritize the implementation of CE.

### Policy and practical implication

The study provides important insight for firms on strategies to implement CE in the Ghanaian C&D industry. The study’s findings suggest firms can effectively implement CE by prioritizing important CE practices based on reduce and recover principles at the design stage. The study also suggests that NGOs, professional bodies, and individual firms committed to supporting firms in the C&D industry to adopt CE practices can organize workshops to train many firms on techniques to implement the identified significant CE practices. Again, the government may have to provide financial support to firms with limited resources to help their transition to CE. Moreover, it is important for the government to develop policies or regulations and to enforce significant CE practices not included in the Ghana Building Code (GS1207).

Finally, the study’s empirical findings in the context of the Ghanaian C&D industry provide important insights on filling the literature gap on strategies for the implementation of CE in emerging economies. In specific response to the non-cohesive framework adopted by the government and firms in the Ghanaian C&D industry (Ahmed et al. 2014; Ayarkwa et al. 2011), the study proposes a cohesive strategic implementation framework that provides a unified approach based on 6R principles for stakeholders in the C&D industry to improve the sustainable production and consumption of resources across the whole life cycle. Thus, the strategic implementation framework informs government, organizations, NGOs, and professional bodies in the Ghanaian C&D industry on the life cycle stage-related CE practices based on 6R principles that need to be prioritized for effective implementation of CE.

## Conclusion

A critical review of existing literature on CE practice implementation highlights the general lack of studies that draws insight from 6R principles to propose a unified approach to CE implementation from the perspective of the whole life cycle in the C&D industry of emerging economies. The study contributes to this gap by characterizing thirty-four CE practices identified from previous studies based on four life cycle stages and 6R principles. A hybrid BWM-GRA was used to prioritize the identified life cycle stages as well as identify the most significant CE practices. Findings from the study suggest firms in other emerging economies need to focus more on CE practices based on reduce and recover principles such as design to improve the energy efficiency of buildings, design to increase the lifespan, design for disassembly, and standardization of designs at the design stage to facilitate the strategic implementation CE. Additionally, the study suggests that firms in Ghana need to focus more on design for disassembly since the other significant CE practices are well applied in the Ghanaian C&D industry. The study also suggests CE practices that should be prioritized at each life cycle stage to facilitate the adoption of the 6R principle in the industry.

The study highlights the significance of both bottom-up and top-down approaches in the implementation of CE practices based on reduce and recover principles at the design stage. The Ghanaian C&D industry and other emerging economies have rapidly been growing in recent years. The lack of CE knowledge risks a significant negative impact on the industry’s environmental performance. Therefore, the proposed strategic implementation framework has the potential to guide stakeholders, particularly management leadership interested in CE practice implementation to develop or amend existing policies, regulations, or laws. The study also encourages stakeholders to support the adoption of environmentally friendly indigenous design practices in modern buildings to facilitate CE practice implementation in the industry. Despite the contributions of the study, it has limitations. The study focused on the Ghanaian C&D industry. Future studies can focus on C&D industries in other emerging economies. Future studies can also use other MCDM tools to validate or compare results from this study. Moreover, future studies can consider engaging different kinds of experts and respondent sizes to enrich insights on this focus.

## Supplementary Information

Below is the link to the electronic supplementary material.

Supplementary file1 (DOCX 293 KB)

## Data Availability

All data generated or analyzed during this study are included in this published article.
