# Supplementary material for: Life cycle stage practices and strategies for circular economy: assessment in construction and demolition industry of an emerging economy
Source: Environ Sci Pollut Res Int. 2022 Jun 24;29(54):82110–21. doi: 10.1007/s11356-022-21470-w (PMC9606091; doi:10.1007/s11356-022-21470-w)
Supplement: Supplementary file 1 — Supplementary file1 (DOCX 293 KB) [file 11356_2022_21470_MOESM1_ESM.docx]

**Supplementary Materials**

**Appendix A**

**Table 6** CE practices at the various life cycle stages

| Life cycle stage |  | CE practices | Sources |
| --- | --- | --- | --- |
| Design stage | PCN01 | Design for reuse of building elements, e.g. columns and doors | (Gangolells et al. 2014) |
|  | PCN02 | Design to improve the energy efficiency of buildings | (Mensah et al. 2017; Asman et al. 2019; Kasim et al. 2020; Rodrigues et al. 2020) |
|  | PCN03 | Design to prevent the generation of waste | (Gorgolewski et al. 2008; European Commission 2014; Ghisellini et al. 2016; Adams et al. 2017; Gálvez-Martos et al. 2018; Mangialardo and Micelli 2018; Wahyu Adi and Wibowo 2020; López Ruiz et al. 2020) |
|  | PCN04 | Make allowance for incentives for CE practices in the contract document | (Huang et al. 2018) |
|  | PCN05 | Design for deconstruction to ensure efficient recovery of materials | (Gorgolewski et al. 2008; Jaillon and Poon 2014; Nußholz et al. 2019; Hossain et al. 2020; O’Grady et al. 2021) |
|  | PCN06 | Design with sustainable local materials such as rammed earth, clay plaster, etc. | Initial survey results |
|  | PCN07 | Design for the adoption of prefabrication | (Tam et al. 2007; Kyrö et al. 2019) |
|  | PCN08 | Design for easy disassembly | (Jaillon and Poon 2014; Adams et al. 2017; Akanbi et al. 2018; Mangialardo and Micelli 2018; Wahyu Adi and Wibowo 2020; Hossain et al. 2020; O’Grady et al. 2021) |
|  | PCN09 | Design to allow for the use of recycled materials | (Smith and Hung 2015; Ünal et al. 2019) |
|  | PCN10 | Engagement of stakeholders to ensure the design conforms with CE principles | (Zimmann et al. 2016; Mahpour 2018) |
|  | PCN11 | Standardization of designs | Initial survey results |
|  | PCN12 | Design to increase the lifespan | (Kasim et al. 2020; Wahyu Adi and Wibowo 2020; Hossain et al. 2020) |
| Construction | CON01 | Proper material storage location | (Arif et al. 2012; Gálvez-Martos et al. 2018) |
|  | CON02 | Adoption of sustainable construction method | (Gálvez-Martos et al. 2018) |
|  | CON03 | Provide incentives for recycling of waste materials on site | (Mahpour 2018) |
|  | CON04 | Resource optimization to minimize excessive use of resources on site | (Adams et al. 2017; Esa et al. 2017; Gálvez-Martos et al. 2018;) |
|  | CON05 | Encourage re-use of materials | (Gálvez-Martos et al. 2018; Wahyu Adi and Wibowo 2020; López Ruiz et al. 2020) |
|  | CON06 | Minimize material stockholding | (Gálvez-Martos et al. 2018; Kasim et al. 2020) |
|  | CON07 | Encourage prefabrication construction | (Adams et al. 2017; Gálvez-Martos et al. 2018; Mangialardo and Micelli 2018; Minunno et al. 2018; Stephan and Athanassiadis 2018; Eberhardt et al. 2020) |
|  | CON08 | Integration of recycled materials | (Adams et al. 2017) |
|  | CON09 | Proper management of construction process | (Ekanayake and Ofori 2004) |
|  | CON10 | Prevent double handling of materials | Initial survey results |
|  | CON11 | Peer review of designs by stakeholders on-site | Initial survey results |
| Operation | OPN01 | Adoption of sustainable repair and maintenance practices | Initial survey results |
|  | OPN02 | Refurbish/renovate with reused/recycle material | Initial survey results |
|  | OPN03 | Evaluate the life cycle performance of redesigned components | (Thomas and Ding 2018; Böckin and Tillman 2019) |
|  | OPN04 | Efficient practices by end-user to reduce CO2 emissions and environmental footprint | (Ünal et al. 2019) |
|  | OPN05 | Efficient use of building to prevent rapid degradation of materials and elements | Initial survey results |
| End of life | EOL01 | Deconstruction to obtain maximum recovery of the components to be used | (Coelho and De Brito 2012; Chau et al. 2017; Adams et al. 2017; Gálvez-Martos et al. 2018) |
|  | EOL02 | Selective demolition | (Adams et al. 2017) |
|  | EOL03 | End of life audits of recovered materials | (Jiménez-Rivero and García-Navarro 2016) |
|  | EOL04 | Adaptive reuse of the whole or part of the redundant structure or material | (Adams et al. 2017; L. A. Akanbi et al. 2018; Gálvez-Martos et al. 2018; Ghisellini et al, 2018a; Huang et al. 2018; L. W. Zhang et al. 2019) |
|  | EOL05 | Recycling of waste materials | (Ding et al. 2016; Hossain et al. 2016; Xiao et al. 2016; Li et al. 2017; Brambilla et al. 2019; Nußholz et al. 2019; Wahyu Adi and Wibowo 2020; Chen et al. 2022) |
|  | EOL06 | Record of the life cycle performance of materials | Initial survey results |

**Appendix B**

**Step 1:** Determination of a set of decision-making criteria. In this step, a set of criteria is chosen for making a decision.

$\left(c_{1},c_{2}....c_{^{n}}\right)$ (1)

**Step 2:** The best (*B*) criteria (most important or most desirable) and the worst criteria (*W*) (least important, least desirable) factors among the approved factors are determined by decision-makers. In this step, the decision-maker selects the best and the worst factor among the set of factors presented in step 1.

**Step 3:** The preference of the best criterion over all the other criteria is determined based on a score scale between 1 and 9, where a score of 1 denotes equal preference between the best criterion and another criterion, and a score of 9 also denotes the extreme preference of the best criterion over the other criterion. The result is the Best-to-Others (BO) vector would be:

$A_{^{B}}=(a_{^{B1}},a_{^{B2}},...,a_{^{Bn}})$ (2)

where $a_{^{Bj}}$ indicates the preference of the best criterion B over criterion j and it can be deduced that .

**Step 4:** Determine the preference of all the other criteria over the worst criterion using a number between 1 and 9 (where 1 is equally important and 9 is extremely more important). The result is the Others-to-Worst (OW) vector would be:

$A_{w}=(a_{1w},a_{2w},\cdots,a_{nw})^{T}$, (3)

where $a_{jw}$indicates the preference of criterion j over the worst criterion w and it can be deduced that$a_{ww}=1$.

**Step 5:** Find the optimal weights.

$\left(w_{1}^{*},w_{2}^{*},w_{3}^{*}......w_{n}^{*}\right)$ (4)

The optimal weights of criteria will satisfy the following requirements: For each pair of $w_{^{B}}/w_{^{j}}$and$w_{^{j}}/w_{^{w}}$, the ideal situation is where $w_{^{B}}/w_{^{j}}=\alpha_{^{Bj}}$ and$w_{^{j}}/w_{^{w}}=\alpha_{jw}$. Therefore, to be as close as possible to the ideal situation, we should find a solution to the absolute differences , for all $j$ is minimized and can be formulated as follows:

(5)

s.t.

$\sum_{j} w_{j}=1,\begin{matrix} & w_{j}\geq0, \end{matrix}$ for all $j$ (6)

Problem (1) can be transferred to the following linear programming problem:

$$\begin{matrix} min & \end{matrix}\xi^{l}$$

s.t.

, for all$j$

, for all$j$

$$\sum_{j} w_{j}=1,\begin{matrix} & w_{j}\geq0, \end{matrix}\begin{matrix} & for\text{ }all\begin{matrix} j & \end{matrix} \end{matrix}$$

After solving problem (2), the optimal weights $\left(w_{1}^{*},w_{2}^{*},w_{3}^{*}......w_{n}^{*}\right)$and $\xi^{l*}$are obtained.

$C.R=\frac{\xi}{C.Index}$ (7)

$\xi^{l*}$can be directly considered as an indicator of the consistency of the comparison system. The closer the value of $\xi^{l*}$ to zero, the higher the consistency is, and thus the more reliable the comparisons become. The consistency index is determined based on the value of BO vector (Rezaei 2015), as shown in Table A1.

**Table 7** Consistency index scale.

| A_Bw_ | 1 | 2 | 3 | 4 | 5 | 6 | 7 | 8 | 9 |
| --- | --- | --- | --- | --- | --- | --- | --- | --- | --- |
| Consistency index | 0.00 | 0.44 | 1.00 | 1.63 | 2.30 | 3.00 | 3.73 | 4.47 | 5.23 |

The Consistency ratio (C.R) is always a value between 0 and 1. Ratios closer to 0 indicate more consistency

**Appendix C**

**Step 1:** The formation of a decision matrix. In the first step, a decision matrix is constructed involving the alternatives $m$and the number of criteria $n$ ($m$*$n$) using the scale below presented in below

$Y=\left[\begin{matrix} y_{1}(1),x_{1}(2),...y_{1}(j) \\ y_{2}(1),x_{2}(2),...y_{2}(j) \\ ... \\ ... \\ y_{i}(1),\mathrm{y}_{i}(2),...y_{i}(j) \end{matrix}\right]$ (1)

Here $x_{m}(n)$ is the value of $i^{th}$ alternative and $j^{th}$ criterion.

**Step 2:** Standardization of the matrix

In section, since experts used different units in measuring the criteria, the obtained matrices are standard into one-unit matrix. In standardizing, three rules are applied:

1. benefit-type criteria (the bigger, the better);
2. cost-type criteria (the smaller, the better); and
3. medium-type (the closer to a specific value, the better).

Based on the type of criterion, the standardized transformation must be chosen using the following Eqs. (2) - (3):

For the benefit-type criteria:

$Y_{i} (\kappa)=\frac{y_{ij} -Min\{y_{ij},i=1,2,...,m\}}{Max\{y_{ij},i=1,2,...,m\}-Min\{y_{ij},i=1,2,...,m\}}$ for $i=1,2,...,m$ (2)

For the cost-type criteria:

$Y_{i} (\kappa)=\frac{y_{ij} -Min\{y_{ij},i=1,2,...,m\}-y_{ij} }{Max\{y_{ij},i=1,2,...,m\}-Min\{y_{ij},i=1,2,...,m\}}$ for $i=1,2,...,m$ (3)

For the medium-type criteria

(4)

for $i=1,2,...,m$and $j=1,2,...,n$.

After using Eqs. (3) - (5) for determining the grey rational generating values, the performance values are normalized between [0,1], such that for criteria $j$of alternative $i$, if the value of $x_{ij}$which has been processed is equivalent to 1 or close to 1 than the value for any other alternative. This means performance of alternative $i$ is the best deal for the criteria $j$. Thus, an alternative will be the best choice/selected if all of its performance values are nearer to or equal to 1.

**Step 3:** The absolute difference between the referential and the compared series can be calculated using

(5)

**Step 4:** Determination of grey relation coefficient.

The grey relation coefficient ξ is calculated using Eq. (5). The Grey relational coefficient is used for determining how nearer $x_{ij}$ is to $x_{0j}$. The larger the grey relational coefficient, the closer $x_{ij}$

for $i=1,2,...,m$and $j=1,2,...,n$ (6)

In Eq. (4) is the grey relational coefficient between $x_{ij}$ is to $x_{0j}$ and

$\Delta_{ij}=Min\{\Delta_{ij},i=1,2,...,...m;j=1,2,...n\}$,

$$\Delta_{ij}=Max\{\Delta_{ij},i=1,2,...,...m;j=1,2,...n\},$$

$\xi$is the distinguishing coefficient $\xi\in[0,1]$. The aim of distinguishing the coefficient is to expand the range of the grey relational coefficient. For instance, if there are three alternatives such as $"a"$, $"b"$and $"c"$. Hence, if $\Delta_{aj}=0.1$, $\Delta_{bj}=0.4$and $\Delta_{cj}=0.9$, its means that for criteria $j$ and alternative $"a"$ is the nearer to the reference sequence. After grey relational generating using Eq. (2) – (4), $\Delta_{max}$will be equal to 1 and $\Delta_{min}$will be equal to 0. The differences between and always change when different coefficients are adopted but do not affect the ranking order and . The distinguishing coefficient can be adjusted by the decision-maker exercising judgment, and different distinguishing coefficients usually produce different results of GRA. In this study distinguishing coefficient was set at 0.5.

**Step 5:** Grey relational grade calculation

After calculating the entire grey relational coefficient the grey relational grade can be then calculated using Eq. (7).

for $i=1,2,...,m$ (7)

So in equation five (5) the grey rational grade is $\Gamma(X_{0},X_{i})$ expressed by $X_{i}$ and $X_{0}$. $w_{j}$ is the weight of criteria $j$which are determined by on decision-makers’ judgment. Again, $\sum_{j=1}^{n} w_{j}\gamma=1$. The grey relational grade indicates the degree of similarity between the comparability sequence and the reference sequence. As mention earlier, each criteria reference sequence denotes the best performance that could be achieved by any among the comparability sequences.

**Appendix D**

***Step 1*:** Determination of set of criteria and alternatives and decision-makers using Eqs. (1) -(3)

$\{c_{1} ,c_{2} ,c_{3} ,...,c_{j} \}$ (2)

$\{a_{1} ,a_{2} ,a_{3} ,...,a_{3} \}$ (3)

$\{D_{1} ,D_{2} ,D_{3} ,...,D_{k} \}$ (4)

***Step 2****:* Construct the decision matrices by each expert:

$\{X^{D_{1}} ,X^{D_{1}} ,X^{D_{1}} ,...,X^{D_{\kappa}} \}$ (5)

***Step 3:*** Application of geometric mean method to integrate the decision matrices ($Y$):

$$Y_{i} =(\prod_{i=1}^{k} X^{D_{i}})^{1/k}$$

***Step 4:*** Using one of the following states (a-c) to normalize the integrate the decision matrix as indicated below:

For the benefit-type criteria:

a. $Y_{i} (\kappa)=\frac{y_{ij} -Min\{y_{ij},i=1,2,...,m\}}{Max\{y_{ij},i=1,2,...,m\}-Min\{y_{ij},i=1,2,...,m\}}$ for $i=1,2,...,m$ (6)

For the cost-type criteria:

b. $Y_{i} (\kappa)=\frac{y_{ij} -Min\{y_{ij},i=1,2,...,m\}-y_{ij} }{Max\{y_{ij},i=1,2,...,m\}-Min\{y_{ij},i=1,2,...,m\}}$ for $i=1,2,...,m$ (7)

For the medium-type criteria

c. (8)

***Step 5*:** After that, the absolute difference between the referential and the compared series is calculated.

(9)

***Step 6***: Grey relation coefficient $\xi$ is calculated using Eqn.(10)

(10)

***Step 7***: At this step, the best and worst criteria are determined by each expert using Eqs. (11) – (12)

$\{B^{e_{1}} ,B^{e_{2}} ,B^{e3} ...,B^{e_{\kappa}} \}$ (11)

$\{w^{e_{1}} ,w^{e_{2}} ,w^{e3} ...,w^{e_{\kappa}} \}$ (12)

***Step 8:*** At this stage, each expert determines the degree of preference of the best criterion to the other criteria.

$\begin{matrix} A_{B}^{e_{1}} =(a_{B_{1}}^{e_{1}} ,a_{B_{2,}}^{e_{1}} a_{B_{3}}^{e_{1}} ...a_{B_{n}}^{e_{1}} ) \\ . \\ . \\ . \\ A_{B}^{e_{\kappa}} =(a_{B_{1}}^{e_{\kappa}} ,a_{B_{2,}}^{e_{\kappa}} a_{B_{3}}^{e_{\kappa}} ...a_{B_{n}}^{e_{\kappa}} ) \end{matrix}$ (13)

***Step 9:*** At this stage, each expert determines the degree of worst criterion to the other criteria.

$\begin{matrix} A_{w}^{e_{1}} =(a_{1w}^{e_{1}} ,a_{2w_{,}}^{e_{1}} a_{3w}^{e_{1}} ...a_{nw}^{e_{1}} )^{T} \\ . \\ . \\ . \\ A_{w}^{e_{\kappa}} =(a_{1w}^{e_{\kappa}} ,a_{2w,}^{e_{\kappa}} a_{3w}^{e_{\kappa}} ...a_{nw}^{e_{\kappa}} )^{T} \end{matrix}$ (14)

***Step 10***: The optimal weight for the criteria weigh for each expert are determined by using Eq. (15)

$$\begin{matrix} min & \end{matrix}\xi^{l}$$

*s.t.*

, for all$j$ (15)

, for all$j$ (16)

$$\sum_{j} w_{j}=1,\begin{matrix} & w_{j}\geq0, \end{matrix}\begin{matrix} & for\text{ }all\begin{matrix} j & \end{matrix} \end{matrix}$$

***Step 11:*** In this section, we use Eq. (17) to aggregate all the weights.

$W_{j} =(\prod_{i=1}^{k} W^{D_{i}})^{1/k}$ (17)

***Step 12:*** The grey relation grade is determined based on Eq. (18) Then, the alternatives are

ranked according to the obtained scores.

$R_{i} =\sum[W_{j} \xi(\kappa)]$ (18)

**Appendix E**

**Table 8** Background nformation of experts

| Expert | Institution | Position |
| --- | --- | --- |
| 1 | Local government | District Engineer |
| 2 | Consultancy firm | Architect |
| 3 | Consultancy firm | Quantity Surveyor |
| 4 | Consultancy firm | Project Manager |
| 5 | Construction firm | Site Engineer |
| 6 | Construction firm | Architect |
| 7 | Real estate development firm | Facility Manager |
| 8 | Academia | Associate Professor |
| 9 | Academia | Associate Professor |
| 10 | Academia | Associate Professor |

**Appendix F**

**Table 9** Most and least important criteria identified by the 1-10 expert (Rezaei et al. 2016)

| Life cycle stage | “Best” criteria identified by the respondent | “Worst” criteria identified by the respondent |
| --- | --- | --- |
| Design | 1,2,3,4,5,6,7,8,9,10 |  |
| Construction |  | 3,9 |
| Operation |  | 1,2,6 |
| End of life |  | 4,5,7,8,10 |

**Table 10** Most important life cycle stage compared with the others (Rezaei et al. 2016)

| Respondent No. | Most important life cycle stage | LCS1 | LCS2 | LCS3 | LCS4 |
| --- | --- | --- | --- | --- | --- |
| 1 | LCS1 | 1 | 2 | 9 | 2 |
| 2 | LCS1 | 1 | 5 | 9 | 2 |
| 3 | LCS1 | 1 | 9 | 6 | 3 |
| 4 | LCS1 | 1 | 3 | 2 | 9 |
| 5 | LCS1 | 1 | 2 | 5 | 7 |
| 6 | LCS1 | 1 | 7 | 8 | 5 |
| 7 | LCS1 | 1 | 4 | 3 | 7 |
| 8 | LCS1 | 1 | 7 | 5 | 8 |
| 9 | LCS1 | 1 | 7 | 4 | 6 |
| 10 | LCS1 | 1 | 6 | 6 | 8 |

**Table 11** The least important life cycle stage compared with the others (Rezaei et al. 2016)

| Respondent number | 1 | 2 | 3 | 4 | 5 | 6 | 7 | 8 | 9 | 10 |
| --- | --- | --- | --- | --- | --- | --- | --- | --- | --- | --- |
| Least important | LCS3 | LCS3 | LCS2 | LCS4 | LCS5 | LCS3 | LCS4 | LCS4 | LCS2 | LCS4 |
| LCS1 | 9 | 9 | 9 | 9 | 7 | 9 | 7 | 8 | 7 | 8 |
| LCS2 | 2 | 5 | 1 | 4 | 6 | 8 | 5 | 5 | 1 | 4 |
| LCS3 | 1 | 1 | 3 | 2 | 5 | 1 | 4 | 3 | 5 | 4 |
| LCS4 | 2 | 8 | 7 | 1 | 1 | 3 | 1 | 1 | 3 | 1 |

**Appendix G**

**Table 12** Pairwise comparison of CE practices by expert 1

| Design Stage (LCS1) | | Construction Stage (LCS2) | | Operation stage (LCS3) | | End of life stage (LCS4) | |
| --- | --- | --- | --- | --- | --- | --- | --- |
| PCN01 | 9 | CON01 | 9 | OPN01 | 9 | EOL01 | 9 |
| PCN02 | 9 | CON02 | 9 | OPN02 | 7 | EOL02 | 9 |
| PCN03 | 9 | CON03 | 9 | OPN03 | 9 | EOL03 | 9 |
| PCN04 | 9 | CON04 | 9 | OPN04 | 9 | EOL04 | 9 |
| PCN05 | 9 | CON05 | 9 | OPN05 | 5 | EOL05 | 9 |
| PCN06 | 9 | CON06 | 1 |  |  | EOL06 | 7 |
| PCN07 | 9 | CON07 | 7 |  |  |  |  |
| PCN08 | 9 | CON08 | 9 |  |  |  |  |
| PCN09 | 9 | CON09 | 9 |  |  |  |  |
| PCN10 | 9 | CON10 | 1 |  |  |  |  |
| PCN11 | 9 | CON11 | 9 |  |  |  |  |
| PCN12 | 9 |  |  |  |  |  |  |

**Table 13** Pairwise comparison of CE practices by expert 2

| Design Stage (LCS1) | | Construction Stage (LCS2) | | Operation stage (LCS3) | | End of life stage (LCS4) | |
| --- | --- | --- | --- | --- | --- | --- | --- |
| PCN01 | 3 | CON01 | 3 | OPN01 | 5 | EOL01 | 3 |
| PCN02 | 9 | CON02 | 7 | OPN02 | 3 | EOL02 | 5 |
| PCN03 | 5 | CON03 | 3 | OPN03 | 5 | EOL03 | 3 |
| PCN04 | 3 | CON04 | 3 | OPN04 | 9 | EOL04 | 9 |
| PCN05 | 7 | CON05 | 9 | OPN05 | 7 | EOL05 | 7 |
| PCN06 | 3 | CON06 | 5 |  |  | EOL06 | 3 |
| PCN07 | 3 | CON07 | 3 |  |  |  |  |
| PCN08 | 9 | CON08 | 3 |  |  |  |  |
| PCN09 | 7 | CON09 | 5 |  |  |  |  |
| PCN10 | 5 | CON10 | 3 |  |  |  |  |
| PCN11 | 9 | CON11 | 3 |  |  |  |  |
| PCN12 | 9 |  |  |  |  |  |  |

**Table 14** Pairwise comparison of CE practices by expert 3

| Design Stage (LCS1) | | Construction Stage (LCS2) | | Operation stage (LCS3) | | End of life stage (LCS4) | |
| --- | --- | --- | --- | --- | --- | --- | --- |
| PCN01 | 9 | CON01 | 5 | OPN01 | 7 | EOL01 | 7 |
| PCN02 | 7 | CON02 | 9 | OPN02 | 7 | EOL02 | 5 |
| PCN03 | 5 | CON03 | 7 | OPN03 | 7 | EOL03 | 9 |
| PCN04 | 3 | CON04 | 9 | OPN04 | 9 | EOL04 | 9 |
| PCN05 | 7 | CON05 | 9 | OPN05 | 9 | EOL05 | 9 |
| PCN06 | 7 | CON06 | 3 |  |  | EOL06 | 5 |
| PCN07 | 7 | CON07 | 5 |  |  |  |  |
| PCN08 | 7 | CON08 | 9 |  |  |  |  |
| PCN09 | 5 | CON09 | 9 |  |  |  |  |
| PCN10 | 5 | CON10 | 3 |  |  |  |  |
| PCN11 | 9 | CON11 | 3 |  |  |  |  |
| PCN12 | 9 |  |  |  |  |  |  |

| Design Stage (LCS1) | | Construction Stage (LCS2) | | Operation stage (LCS3) | | End of life stage (LCS4) | |
| --- | --- | --- | --- | --- | --- | --- | --- |
| PCN01 | 1 | CON01 | 1 | OPN01 | 1 | EOL01 | 3 |
| PCN02 | 9 | CON02 | 7 | OPN02 | 1 | EOL02 | 3 |
| PCN03 | 3 | CON03 | 5 | OPN03 | 1 | EOL03 | 3 |
| PCN04 | 1 | CON04 | 5 | OPN04 | 1 | EOL04 | 1 |
| PCN05 | 1 | CON05 | 7 | OPN05 | 1 | EOL05 | 1 |
| PCN06 | 3 | CON06 | 7 |  |  | EOL06 | 1 |
| PCN07 | 5 | CON07 | 5 |  |  |  |  |
| PCN08 | 5 | CON08 | 7 |  |  |  |  |
| PCN09 | 3 | CON09 | 7 |  |  |  |  |
| PCN10 | 5 | CON10 | 5 |  |  |  |  |
| PCN11 | 5 | CON11 | 3 |  |  |  |  |
| PCN12 | 5 |  |  |  |  |  |  |

**Table 15** Pairwise comparison of CE practices by expert 4

**Table 16** Pairwise comparison of CE practices by expert 5

| Design Stage (LCS1) | | Construction Stage (LCS2) | | Operation stage (LCS3) | | End of life stage (LCS4) | |
| --- | --- | --- | --- | --- | --- | --- | --- |
| PCN01 | 9 | CON01 | 7 | OPN01 | 9 | EOL01 | 5 |
| PCN02 | 9 | CON02 | 9 | OPN02 | 7 | EOL02 | 5 |
| PCN03 | 5 | CON03 | 1 | OPN03 | 9 | EOL03 | 5 |
| PCN04 | 1 | CON04 | 5 | OPN04 | 7 | EOL04 | 7 |
| PCN05 | 7 | CON05 | 7 | OPN05 | 9 | EOL05 | 5 |
| PCN06 | 7 | CON06 | 7 |  |  | EOL06 | 5 |
| PCN07 | 5 | CON07 | 5 |  |  |  |  |
| PCN08 | 9 | CON08 | 5 |  |  |  |  |
| PCN09 | 3 | CON09 | 7 |  |  |  |  |
| PCN10 | 7 | CON10 | 5 |  |  |  |  |
| PCN11 | 9 | CON11 | 9 |  |  |  |  |
| PCN12 | 9 |  |  |  |  |  |  |

| Design Stage (LCS1) | | Construction Stage (LCS2) | | Operation stage (LCS3) | | End of life stage (LCS4) | |
| --- | --- | --- | --- | --- | --- | --- | --- |
| PCN01 | 9 | CON01 | 5 | OPN01 | 7 | EOL01 | 9 |
| PCN02 | 9 | CON02 | 9 | OPN02 | 5 | EOL02 | 7 |
| PCN03 | 7 | CON03 | 9 | OPN03 | 7 | EOL03 | 9 |
| PCN04 | 9 | CON04 | 9 | OPN04 | 9 | EOL04 | 9 |
| PCN05 | 9 | CON05 | 9 | OPN05 | 9 | EOL05 | 9 |
| PCN06 | 9 | CON06 | 3 |  |  | EOL06 | 7 |
| PCN07 | 5 | CON07 | 5 |  |  |  |  |
| PCN08 | 7 | CON08 | 7 |  |  |  |  |
| PCN09 | 9 | CON09 | 7 |  |  |  |  |
| PCN10 | 7 | CON10 | 5 |  |  |  |  |
| PCN11 | 7 | CON11 | 9 |  |  |  |  |
| PCN12 | 5 |  |  |  |  |  |  |

**Table 17** Pairwise comparison of CE practices by expert 6

| Design Stage (LCS1) | | Construction Stage (LCS2) | | Operation stage (LCS3) | | End of life stage (LCS4) | |
| --- | --- | --- | --- | --- | --- | --- | --- |
| PCN01 | 9 | CON01 | 7 | OPN01 | 5 | EOL01 | 7 |
| PCN02 | 9 | CON02 | 7 | OPN02 | 5 | EOL02 | 7 |
| PCN03 | 9 | CON03 | 7 | OPN03 | 7 | EOL03 | 7 |
| PCN04 | 9 | CON04 | 7 | OPN04 | 7 | EOL04 | 7 |
| PCN05 | 9 | CON05 | 7 | OPN05 | 7 | EOL05 | 7 |
| PCN06 | 9 | CON06 | 7 |  |  | EOL06 | 5 |
| PCN07 | 9 | CON07 | 5 |  |  |  |  |
| PCN08 | 9 | CON08 | 5 |  |  |  |  |
| PCN09 | 7 | CON09 | 5 |  |  |  |  |
| PCN10 | 7 | CON10 | 7 |  |  |  |  |
| PCN11 | 7 | CON11 | 9 |  |  |  |  |
| PCN12 | 9 |  |  |  |  |  |  |

**Table 18** Pairwise comparison of CE practices by expert 7

**Table 19** Pairwise comparison of CE practices by expert 8

| Design Stage (LCS1) | | Construction Stage (LCS2) | | Operation stage (LCS3) | | End of life stage (LCS4) | |
| --- | --- | --- | --- | --- | --- | --- | --- |
| PCN01 | 7 | CON01 | 9 | OPN01 | 3 | EOL01 | 5 |
| PCN02 | 3 | CON02 | 7 | OPN02 | 5 | EOL02 | 3 |
| PCN03 | 3 | CON03 | 7 | OPN03 | 7 | EOL03 | 1 |
| PCN04 | 3 | CON04 | 9 | OPN04 | 9 | EOL04 | 5 |
| PCN05 | 7 | CON05 | 7 | OPN05 | 7 | EOL05 | 3 |
| PCN06 | 7 | CON06 | 9 |  |  | EOL06 | 5 |
| PCN07 | 7 | CON07 | 9 |  |  |  |  |
| PCN08 | 7 | CON08 | 7 |  |  |  |  |
| PCN09 | 7 | CON09 | 9 |  |  |  |  |
| PCN10 | 7 | CON10 | 7 |  |  |  |  |
| PCN11 | 7 | CON11 | 9 |  |  |  |  |
| PCN12 | 7 |  |  |  |  |  |  |

| Design Stage (LCS1) | | Construction Stage (LCS2) | | Operation stage (LCS3) | | End of life stage (LCS4) | |
| --- | --- | --- | --- | --- | --- | --- | --- |
| PCN01 | 9 | CON01 | 9 | OPN01 | 3 | EOL01 | 7 |
| PCN02 | 9 | CON02 | 9 | OPN02 | 7 | EOL02 | 7 |
| PCN03 | 9 | CON03 | 9 | OPN03 | 7 | EOL03 | 7 |
| PCN04 | 9 | CON04 | 9 | OPN04 | 7 | EOL04 | 7 |
| PCN05 | 9 | CON05 | 9 | OPN05 | 7 | EOL05 | 7 |
| PCN06 | 9 | CON06 | 9 |  |  | EOL06 | 7 |
| PCN07 | 9 | CON07 | 9 |  |  |  |  |
| PCN08 | 9 | CON08 | 9 |  |  |  |  |
| PCN09 | 9 | CON09 | 9 |  |  |  |  |
| PCN10 | 9 | CON10 | 9 |  |  |  |  |
| PCN11 | 8 | CON11 | 9 |  |  |  |  |
| PCN12 | 9 |  |  |  |  |  |  |

**Table 20** Pairwise comparison of CE practices by expert 9

| Design Stage (LCS1) | | Construction Stage (LCS2) | | Operation stage (LCS3) | | End of life stage (LCS4) | |
| --- | --- | --- | --- | --- | --- | --- | --- |
| PCN01 | 5 | CON01 | 5 | OPN01 | 5 | EOL01 | 7 |
| PCN02 | 3 | CON02 | 9 | OPN02 | 7 | EOL02 | 7 |
| PCN03 | 7 | CON03 | 3 | OPN03 | 5 | EOL03 | 5 |
| PCN04 | 7 | CON04 | 5 | OPN04 | 5 | EOL04 | 5 |
| PCN05 | 5 | CON05 | 7 | OPN05 | 5 | EOL05 | 9 |
| PCN06 | 5 | CON06 | 3 |  |  | EOL06 | 5 |
| PCN07 | 9 | CON07 | 3 |  |  |  |  |
| PCN08 | 3 | CON08 | 5 |  |  |  |  |
| PCN09 | 7 | CON09 | 5 |  |  |  |  |
| PCN10 | 5 | CON10 | 9 |  |  |  |  |
| PCN11 | 3 | CON11 | 5 |  |  |  |  |
| PCN12 | 5 |  |  |  |  |  |  |

**Table 21** Pairwise comparison of CE practices by expert 10

**Appendix H**

**Table 22** Normalization of CE practices using Grey-Relational Analysis model.

| Design Stage (LCS1) | | Construction Stage (LCS2) | | Operation stage (LCS3) | | End of life stage (LCS4) | |
| --- | --- | --- | --- | --- | --- | --- | --- |
| PCN01 | 0.727 | CON01 | 0.214 | OPN01 | 0.000 | EOL01 | 0.333 |
| PCN02 | 1.000 | CON02 | 1.000 | OPN02 | 0.000 | EOL02 | 0.556 |
| PCN03 | 0.364 | CON03 | 0.214 | OPN03 | 0.556 | EOL03 | 0.556 |
| PCN04 | 0.000 | CON04 | 0.571 | OPN04 | 1.000 | EOL04 | 0.000 |
| PCN05 | 0.727 | CON05 | 0.929 | OPN05 | 0.667 | EOL05 | 0.111 |
| PCN06 | 0.636 | CON06 | 0.000 |  |  | EOL06 | 1.000 |
| PCN07 | 0.636 | CON07 | 0.071 |  |  |  |  |
| PCN08 | 0.909 | CON08 | 0.429 |  |  |  |  |
| PCN09 | 0.545 | CON09 | 0.643 |  |  |  |  |
| PCN10 | 0.545 | CON10 | 0.000 |  |  |  |  |
| PCN11 | 0.864 | CON11 | 0.500 |  |  |  |  |
| PCN12 | 1.000 |  |  |  |  |  |  |

**Table 23** Grey Relations Coefficient of the Life cycle stages

| Design Stage (LCS1) | | Construction Stage (LCS2) | | Operation stage (LCS3) | | End of life stage (LCS4) | |
| --- | --- | --- | --- | --- | --- | --- | --- |
| PCN01 | 0.647 | CON01 | 0.389 | OPN01 | 0.333 | EOL01 | 0.429 |
| PCN02 | 1.000 | CON02 | 1.000 | OPN02 | 0.333 | EOL02 | 0.529 |
| PCN03 | 0.440 | CON03 | 0.389 | OPN03 | 0.529 | EOL03 | 0.529 |
| PCN04 | 0.333 | CON04 | 0.538 | OPN04 | 1.000 | EOL04 | 0.333 |
| PCN05 | 0.647 | CON05 | 0.875 | OPN05 | 0.600 | EOL05 | 1.000 |
| PCN06 | 0.579 | CON06 | 0.333 |  |  | EOL06 | 1.000 |
| PCN07 | 0.579 | CON07 | 0.350 |  |  |  |  |
| PCN08 | 0.846 | CON08 | 0.467 |  |  |  |  |
| PCN09 | 0.524 | CON09 | 0.583 |  |  |  |  |
| PCN10 | 0.524 | CON10 | 0.333 |  |  |  |  |
| PCN11 | 0.786 | CON11 | 0.500 |  |  |  |  |
| PCN12 | 1.000 |  |  |  |  |  |  |

**Table 24** Grey Relations Grade of the Life cycle stage

| Design Stage (LCS1) | | Construction Stage (LCS2) | | Operation stage (LCS3) | | End of life stage (LCS4) | |
| --- | --- | --- | --- | --- | --- | --- | --- |
| PCN01 | 0.373 | CON01 | 0.059 | OPN01 | 0.045 | EOL01 | 0.059 |
| PCN02 | 0.576 | CON02 | 0.152 | OPN02 | 0.045 | EOL02 | 0.073 |
| PCN03 | 0.254 | CON03 | 0.059 | OPN03 | 0.071 | EOL03 | 0.073 |
| PCN04 | 0.192 | CON04 | 0.082 | OPN04 | 0.134 | EOL04 | 0.046 |
| PCN05 | 0.373 | CON05 | 0.133 | OPN05 | 0.080 | EOL05 | 0.137 |
| PCN06 | 0.334 | CON06 | 0.051 |  |  | EOL06 | 0.137 |
| PCN07 | 0.334 | CON07 | 0.053 |  |  |  |  |
| PCN08 | 0.488 | CON08 | 0.071 |  |  |  |  |
| PCN09 | 0.302 | CON09 | 0.089 |  |  |  |  |
| PCN10 | 0.302 | CON10 | 0.051 |  |  |  |  |
| PCN11 | 0.453 | CON11 | 0.076 |  |  |  |  |

**References**

Adams KT, Osmani M, Thorpe T, Thornback J (2017) Circular economy in construction: current awareness, challenges and enablers. Proc Inst Civ Eng - Waste Resour Manag 170:15–24. https://doi.org/10.1680/jwarm.16.00011

Akanbi LA, Oyedele LO, Akinade OO, et al (2018) Salvaging building materials in a circular economy: A BIM-based whole-life performance estimator. Resour Conserv Recycl. https://doi.org/10.1016/j.resconrec.2017.10.026

Arif M, Bendi D, Toma-Sabbagh T, Sutrisna M (2012) Construction waste management in India: An exploratory study. Constr Innov. https://doi.org/10.1108/14714171211215912

Asman GE, Kissi E, Agyekum K, et al (2019) Critical components of Environmentally Sustainable Buildings Design Practices of office buildings in Ghana. J Build Eng. https://doi.org/10.1016/j.jobe.2019.100925

Böckin D, Tillman AM (2019) Environmental assessment of additive manufacturing in the automotive industry. J Clean Prod. https://doi.org/10.1016/j.jclepro.2019.04.086

Brambilla G, Lavagna M, Vasdravellis G, Castiglioni CA (2019) Environmental benefits arising from demountable steel-concrete composite floor systems in buildings. Resour Conserv Recycl. https://doi.org/10.1016/j.resconrec.2018.10.014

Chau CK, Xu JM, Leung TM, Ng WY (2017) Evaluation of the impacts of end-of-life management strategies for deconstruction of a high-rise concrete framed office building. Appl Energy. https://doi.org/10.1016/j.apenergy.2016.01.019

Chen R, Li L, Yang K, et al (2022) Quantitative methods for predicting underground construction waste considering reuse and recycling. Environ Sci Pollut Res. https://doi.org/10.1007/s11356-021-15858-3

Coelho A, De Brito J (2012) Influence of construction and demolition waste management on the environmental impact of buildings. Waste Manag. https://doi.org/10.1016/j.wasman.2011.11.011

Ding T, Xiao J, Tam VWY (2016) A closed-loop life cycle assessment of recycled aggregate concrete utilization in China. Waste Manag. https://doi.org/10.1016/j.wasman.2016.05.031

Eberhardt LCM, Birkved M, Birgisdottir H (2020) Building design and construction strategies for a circular economy. Archit Eng Des Manag. https://doi.org/10.1080/17452007.2020.1781588

Ekanayake LL, Ofori G (2004) Building waste assessment score: Design-based tool. Build Environ. https://doi.org/10.1016/j.buildenv.2004.01.007

Esa MR, Halog A, Rigamonti L (2017) Developing strategies for managing construction and demolition wastes in Malaysia based on the concept of circular economy. J Mater Cycles Waste Manag 19:1144–1154. https://doi.org/10.1007/s10163-016-0516-x

European Commission (2014) Communication from the Commission - Towards a circular economy: A zero waste programme for Europe. Eur Comm

Gálvez-Martos JL, Styles D, Schoenberger H, Zeschmar-Lahl B (2018) Cited in Construction and demolition waste best management practice in Europe. Resour Conserv Recycl. https://doi.org/10.1016/j.resconrec.2018.04.016

Gangolells M, Casals M, Forcada N, Macarulla M (2014) Analysis of the implementation of effective waste management practices in construction projects and sites. Resour Conserv Recycl. https://doi.org/10.1016/j.resconrec.2014.10.006

Ghisellini P, Cialani C, Ulgiati S (2016) A review on circular economy: The expected transition to a balanced interplay of environmental and economic systems. J Clean Prod. https://doi.org/10.1016/j.jclepro.2015.09.007

Ghisellini P, Ripa M, Ulgiati S (2018) Exploring environmental and economic costs and benefits of a circular economy approach to the construction and demolition sector. A literature review. J Clean Prod. https://doi.org/10.1016/j.jclepro.2017.11.207

Gorgolewski M, Straka V, Edmonds J, Sergio-Dzoutzidis C (2008) Designing buildings using reclaimed steel components. J Green Build. https://doi.org/10.3992/jgb.3.3.97

Hossain MU, Ng ST, Antwi-Afari P, Amor B (2020) Circular economy and the construction industry: Existing trends, challenges and prospective framework for sustainable construction. Renew Sustain Energy Rev 130:109948. https://doi.org/10.1016/j.rser.2020.109948

Hossain MU, Poon CS, Lo IMC, Cheng JCP (2016) Comparative environmental evaluation of aggregate production from recycled waste materials and virgin sources by LCA. Resour Conserv Recycl. https://doi.org/10.1016/j.resconrec.2016.02.009

Huang B, Wang X, Kua H, et al (2018) Construction and demolition waste management in China through the 3R principle. Resour Conserv Recycl. https://doi.org/10.1016/j.resconrec.2017.09.029

Jaillon L, Poon CS (2014) Life cycle design and prefabrication in buildings: A review and case studies in Hong Kong. Autom Constr. https://doi.org/10.1016/j.autcon.2013.09.006

Jiménez-Rivero A, García-Navarro J (2016) Indicators to Measure the Management Performance of End-of-Life Gypsum: From Deconstruction to Production of Recycled Gypsum. Waste and Biomass Valorization. https://doi.org/10.1007/s12649-016-9561-x

Kasim I, Sipan I, Daud Z, et al (2020) Implementation of Environmental Sustainability Strategies for Real Estate Development in Developing Nations: An Empirical Study from Ghana. Int J Real Estate Stud

Kyrö R, Jylhä T, Peltokorpi A (2019) Embodying circularity through usable relocatable modular buildings. Facilities. https://doi.org/10.1108/F-12-2017-0129

Li J, Xia R, Li J, Chen G (2017) Environmental Impact Assessment of Construction and Demolition Waste Recycling in Shenzhen. In: Proceedings of the 20th International Symposium on Advancement of Construction Management and Real Estate. Springer Singapore, Singapore, pp 1101–1110

López Ruiz LA, Roca Ramón X, Gassó Domingo S (2020) The circular economy in the construction and demolition waste sector – A review and an integrative model approach. J Clean Prod 248:119238. https://doi.org/10.1016/j.jclepro.2019.119238

Mahpour A (2018) Prioritizing barriers to adopt circular economy in construction and demolition waste management. Resour Conserv Recycl. https://doi.org/10.1016/j.resconrec.2018.01.026

Malmqvist T, Nehasilova M, Moncaster A, et al (2018) Design and construction strategies for reducing embodied impacts from buildings – Case study analysis. Energy Build. https://doi.org/10.1016/j.enbuild.2018.01.033

Mangialardo A, Micelli E (2018) Rethinking the Construction Industry Under the Circular Economy: Principles and Case Studies. In: Green Energy and Technology. pp 333–344

Mensah M, Adu T, Atta G, Junior A (2017) Exploration of Architect perception on energy -efficient design decisions for Ghanaian building industry. Am J Eng Res 44–52

Minunno R, O’Grady T, Morrison GM, et al (2018) Strategies for applying the circular economy to prefabricated buildings. Buildings. https://doi.org/10.3390/buildings8090125

Nußholz JLK, Nygaard Rasmussen F, Milios L (2019) Circular building materials: Carbon saving potential and the role of business model innovation and public policy. Resour Conserv Recycl. https://doi.org/10.1016/j.resconrec.2018.10.036

O’Grady T, Minunno R, Chong HY, Morrison GM (2021) Design for disassembly, deconstruction and resilience: A circular economy index for the built environment. Resour Conserv Recycl. https://doi.org/10.1016/j.resconrec.2021.105847

Rezaei J (2015) Best-worst multi-criteria decision-making method. Omega (United Kingdom) 53:49–57. https://doi.org/10.1016/j.omega.2014.11.009

Rezaei J, Nispeling T, Sarkis J, Tavasszy L (2016) A supplier selection life cycle approach integrating traditional and environmental criteria using the best worst method. J Clean Prod 135:577–588. https://doi.org/10.1016/j.jclepro.2016.06.125

Rodrigues F, Silva-Afonso A, Pinto A, et al (2020) Increasing water and energy efficiency in university buildings: a case study. Environ Sci Pollut Res. https://doi.org/10.1007/s11356-019-04990-w

Smith S, Hung PY (2015) A novel selective parallel disassembly planning method for green design. J Eng Des. https://doi.org/10.1080/09544828.2015.1045841

Stephan A, Athanassiadis A (2018) Towards a more circular construction sector: Estimating and spatialising current and future non-structural material replacement flows to maintain urban building stocks. Resour Conserv Recycl. https://doi.org/10.1016/j.resconrec.2017.09.022

Tam VWY, Tam CM, Zeng SX, Ng WCY (2007) Towards adoption of prefabrication in construction. Build Environ. https://doi.org/10.1016/j.buildenv.2006.10.003

Thomas D, Ding G (2018) Comparing the performance of brick and timber in residential buildings – The case of Australia. Energy Build. https://doi.org/10.1016/j.enbuild.2017.10.094

Ünal E, Urbinati A, Chiaroni D (2019) Managerial practices for designing circular economy business models: The case of an Italian SME in the office supply industry. J Manuf Technol Manag. https://doi.org/10.1108/JMTM-02-2018-0061

Wahyu Adi TJ, Wibowo P (2020) Application of circular economy in the Indonesia construction industry. IOP Conf Ser Mater Sci Eng 849:. https://doi.org/10.1088/1757-899X/849/1/012049

Xiao J, Ma Z, Ding T (2016) Reclamation chain of waste concrete: A case study of Shanghai. Waste Manag. https://doi.org/10.1016/j.wasman.2015.09.018

Zhang LW, Sojobi AO, Kodur VKR, Liew KM (2019) Effective utilization and recycling of mixed recycled aggregates for a greener environment. J Clean Prod. https://doi.org/10.1016/j.jclepro.2019.07.075

Zimmann R, O’Brien O, Hargrave J, Morrell M (2016) The Circular Economy in the Built Environment. Callifornia Acad Sci San Fr USA 1–93
